# ACCIDENTAL POISONING IN CHILDREN AND ADOLESCENTS ADMITTED TO A REFERRAL TOXICOLOGY DEPARTMENT OF A BRAZILIAN EMERGENCY HOSPITAL

**DOI:** 10.1590/1984-0462/2020/38/2018096

**Published:** 2019-11-25

**Authors:** Luciana Vilaça, Fernando Madalena Volpe, Roberto Marini Ladeira

**Affiliations:** a Fundação Hospitalar do Estado de Minas Gerais, Belo Horizonte, MG, Brazil.

**Keywords:** Poisoning, Children, Adolescents, Accident, Risk factors, Intoxicação, Criança, Adolescente, Acidente, Fatores de risco

## Abstract

**Objective:**

To describe the profile of children and adolescents admitted for exogenous unintentional poisoning in the emergency room and analyze factors associated with subsequent in-hospital admissions.

**Methods:**

This is a cross-sectional study based on hospital records of all subjects up to 19 years-old admitted in 2013 at a specialized toxicology service on a major public emergency hospital due to unintentional intoxication (as reported). Accidents with poisonous animals and insects were excluded. Percentages and frequencies were calculated for the qualitative variables, and measures of central tendency and dispersion for the continuous quantitative variables. Multivariate analysis was performed using binary logistic regression to identify variables associated with subsequent in-hospital admissions.

**Results:**

In 2013, 353 cases were reported. Poisonings were more frequent in children 0-4 years-old (72.5%) and in boys (55%). The vast majority was of dwellers of the Metropolitan Region of Belo Horizonte (83%), and 90% of the accidental poisonings occurred at home. 82.7% of the poisonings occurred by oral ingestion, especially of medicinal (36.5%) and cleaning products (29.4% of all poisonings). Only 12.2% of the cases resulted in hospitalization, and only one resulted in death. Residing outside Belo Horizonte (OR=5.20 [95%CI 2.37-11.44]) and poisoning by two or more products (OR=4.29 [95%CI 1.33-13.82]) were considered risk factors for hospitalization.

**Conclusions:**

Accidental poisonings occurred most frequently by ingestion of household medications and cleaning products, especially among children under 4 years-old. Preventive strategies should be primarily directed for this prevalent profile.

## RESUMO

**Objetivo:**

Descrever o perfil dos atendimentos de crianças e adolescentes vítimas de intoxicações exógenas acidentais e os fatores associados às internações hospitalares.

**Métodos:**

Foi realizado um estudo transversal com base na revisão dos registros de todas as intoxicações acidentais de indivíduos com até 19 anos de idade, atendidos no setor de toxicologia de um hospital público de referência em 2013, excluídos os acidentes com animais peçonhentos e insetos. A intencionalidade da intoxicação foi baseada nos relatos. Foram calculadas percentagens e frequências para as variáveis qualitativas, e medidas de tendência central e de dispersão das variáveis quantitativas contínuas. Foi realizada análise múltipla, utilizando regressão logística binária para identificar as variáveis associadas à internação hospitalar das vítimas atendidas.

**Resultados:**

Em 2013, foram identificados 353 atendimentos em crianças e adolescentes. A faixa etária mais prevalente foi a de zero a quatro anos (72,5%), e predominaram indivíduos do sexo masculino (55%). A maioria dos atendimentos foi de pacientes residentes na região metropolitana (83%). Noventa por cento das intoxicações ocorreram nos domicílios; 82,7% se deram pela via oral, especialmente por medicamentos (36,5%) e produtos de limpeza (29,4% de todas as intoxicações). Resultaram em internações 12,2% dos casos, ocorrendo um único óbito. As variáveis associadas à internação foram: residir fora do município sede (razão de chances [OR]=5,20; intervalo de confiança de 95% [IC95%] 2,37-11,44) e o envolvimento de mais do que uma substância na intoxicação (OR=4,29; IC95% 1,33-13,82).

**Conclusões:**

O ambiente doméstico é o principal local em que ocorrem as intoxicações em crianças e adolescentes, especialmente por ingestão de medicamentos e produtos de limpeza e abaixo de quatro anos de idade. Esses achados justificam a priorização de ações preventivas direcionadas para esse perfil de acidentes.

## INTRODUCTION

Accidental poisoning is a global health issue among children and adolescents, with approximately 45 thousand deaths per year and an incidence of 1.8 per 100 thousand inhabitants.^1^ Among 15- to 19-year-olds, it represented the 13th cause of death worldwide in 2014.^1^ Moreover, poisoning leads to a substantial number of hospitalizations^2^.

In 2013, half of the 28,419 poisonings notified to the National Poison Data System of Fiocruz (*Sistema Nacional de Informações Tóxico-Farmacológicas da Fiocruz* - Sinitox^3^) occurred in individuals under 20 years of age, with 29% of them being 1- to 4-year-olds - the age group with the highest incidence. Death by accidental poisoning in children and adolescents is less common than by intentional poisoning. In 2012, out of the 392 deaths registered, 40 were accidental (non-intentional), and only 12 happened in the age group 0-19 years. In a non-specialized emergency care unit in the inland of Minas Gerais, poisoning among individuals under 20 years of age represented 27.7% of cases.^4^

Children, particularly preschoolers, stay a significant part of their time at home, where risk exposure is associated with the access to poisonous substances and medicines.^5^ Caregivers’ lack of knowledge about the toxicity of agents, the inattention to risks, and the lack of supervision contribute to the occurrence of accidental poisoning in childhood.^6^ ^,^ ^7^ ^,^ ^8^ In addition, the improper storage of cleaning products and medicines increases the risk exposure for children at home.^6^ ^,^ ^7^ ^,^ ^8^

Relationships between determinants and outcomes in accidental poisoning during childhood and adolescence change according to the study location. For instance, the type of toxic agent and the ease of access to health services are closely related to the place of residence.^1^ Therefore, assessing the epidemiology of accidental poisoning in different scenarios and regions is necessary to increase the specificity of preventive strategies.

Recent Brazilian studies that specifically describe the epidemiology of accidental acute poisoning in children and adolescents are scarce. Two studies - in Rio de Janeiro^9^ and Rio Grande do Sul^5^ - reported the poisoning profile in children under 6 years of age, both with data collected more than 5 years ago. Two other studies conducted in Brazil addressed accidental poisoning; the first, held in Maringá, Paraná,^10^ ^,^ ^11^ described the profile of individuals up to 14 years; and the other, in Cuiabá, Mato Grosso, included children and young adults.^12^ All national studies report a preponderance of accidents at home, on males, and individuals under four years of age, involving mainly medicines and cleaning products.

This study aimed to investigate the profile of children and adolescent victims of accidental exogenous poisoning treated at a referral toxicology department in Minas Gerais, as well as the factors associated with hospitalization.

## METHOD

This is a cross-sectional study that included all individuals aged zero to 19 years treated at the Toxicology Department of Hospital João XXIII (Belo Horizonte, Minas Gerais), a public state hospital, and diagnosed with accidental exogenous poisoning from January to December 2013. This unit is a public state reference in poisoning. We excluded from the study individuals with intentional exogenous poisoning and accidents caused by venomous and non-venomous animals. Patient or family reports recorded by a health professional determined the intentionality of poisoning. The data source was the patient treatment form of the Toxicology Department. Each treatment performed by the department has one of these forms, usually filled by the medical staff throughout the patient’s hospital stay. In specific cases in which the information on these forms was ambiguous or insufficient, especially regarding the length of stay, we accessed the patient’s electronic medical records to confirm the data.

We adopted the following variables: age (in years); gender; date of the accident; date of the treatment; area of residence (urban or rural); city of residence; route of exposure (ingestion, dermal absorption, inhalation, other); active ingredients of the substance; commercial names of the products; hospitalization (defined as length of stay greater than or equal to 24 hours - yes/no); date of discharge; length of stay; case progression (discharge, death, other).

In the descriptive analyses of treatments, we calculated percentages and frequencies for qualitative variables and measures of central tendency and dispersion for continuous quantitative variables. We compared means and percentages using Student’s *t*-test and Pearson’s chi-square test (or Fisher’s exact test), with a 5% significance level. We conducted a multivariate analysis to identify the variables associated with hospitalization of the victims treated using binary logistic regression and included in the model variables that reached a p-value<0.20 in the univariate analysis with the backward strategy. For these analyses, the dependent variable was hospitalization (dichotomous) and the explanatory variables tested were gender (male or female), age group (up to three years; older than three years), place of residence (Belo Horizonte or other), local of exposure (home or other), number of substances (one, two, or more), substance type (medicines or other), and route of exposure (ingestion or other).

Data were collected without storing information that could allow the individual identification of any participant. The Research Ethics Committees of Fundação Hospitalar do Estado de Minas Gerais (FHEMIG), Report No. 491,927, and Universidade Federal de Minas Gerais (UFMG), Report No. 53476915.5.0000.5149, approved this project.

## RESULTS

In 2013, the Toxicology Department treated 5,013 patients, of which 1,174 belonged to the age group up to 19 years. We found 353 cases of accidental exogenous poisoning in children and adolescents, excluding accidents with venomous animals and insects (n=620; 52.7%), events identified as suicide attempts (n=33; 2.8%), and other diagnoses (n=168; 14.3%).

Table 1 presents the characteristics of these treatments. Most patients lived in Belo Horizonte (70%), while the remaining individuals of the sample resided in other cities from the metropolitan area and other regions of the state. Regarding the area of residence of the victims, a high proportion of them lived in urban areas - 94.9% of cases. Most poisoning cases occurred at the victim’s home (90.1%).

**Table 1:** Characteristics of patients treated for accidental in the Toxicology Department of the Hospital João XXIII, Belo Horizonte, Minas Gerais, Brazil, in 2013 (n=353).

Characteristics	n	%
Gender		
Male	194	(55.0)
Age (years)		
< 1	16	(4.5)
1	92	(26.1)
2	67	(19.0)
3	47	(13.3)
4	34	(9.6)
5 to 12	51	(14.4)
13 to 19	46	(13.0)
Route of exposure		
Ingestion	292	(82.7)
Inhalation	19	(5.4)
Dermal absorption	18	(5.1)
Other	1	(0.3)
Not informed	23	(6.5)
Products		
Medicine	128	(36.3)
Chemical and cleaning product	105	(29.7)
Pesticide	39	(11.1)
Toxic plant	14	(4.0)
Cosmetic product	7	(2.0)
Other	10	(2.8)
Not informed	50	(14.2)
Number of products		
1	283	(80.5)
2	17	(5.5)
3	7	(2.8)
Not informed	46	(12.2)
Hospitalization		
Yes	43	(12.2)
Progression		
Discharge	348	(98.6)
Left hospital against medical advice	3	(0.8)
Death	1	(0.3)

The incidence of males was slightly higher (54.9%). The age with the highest prevalence among victims of accidental exogenous poisoning was one year-old, with 26.1% of cases; while <1-year-old patients had the lowest representativeness, with 4.5% of cases. Children aged 0-4 years accounted for 72.6% of cases.

Analyzing the age distribution according to gender, we found a predominance of males aged up to 2 years and balance between genders in the 3-12-year-old group. However, among adolescents, the predominance of females in the 13-19-year-old group was clear (60.9%).

With respect to routes of exposure, ingestion (82.7%) was the most frequent and dermal absorption (5.1%), the least. Most (80.5%) poisonings had only one product identified as the cause. Poisoning by more than two products simultaneously was uncommon (2.8%).

Among the substances more often involved in exogenous poisoning in children and adolescents, medicines were the most relevant, followed by chemicals/cleaning products and pesticides (Table 2). Pesticides also covered rodenticides and acaricides; and “other” included: food, alcoholic beverages, jellyfish, lime, cement, nicotine, and tetrahydrocannabinol.

**Table 2:** Distribution of accidental exogenous poisoning in the population aged zero to 19 years, according to product category and age of the victim treated at the Hospital João XXIII in Belo Horizonte, Minas Gerais, Brazil, 2013 (n=353).

Product	Age group							
	< 1 year	1 year	2 years	3 years	4 years	5 to 12 years	13 to 19 years	Total
	n (%)	n (%)	n (%)	n (%)	n (%)	n (%)	n (%)	n (%)
Medicines	5 (31.2)	19 (20.7)	23 (34.3)	25 (53.2)	20 (58.8)	19 (37.3)	17 (37.0)	128 (36.3)
Chemicals and cleaning products	6 (37.5)	36 (39.0)	23 (34.3)	8 (17.0)	8 (23.5)	11 (21.5)	13 (28.2)	105 (29.7)
Pesticides	-	19 (20.7)	10 (14.9)	7 (14.9)	1 (2.9)	1 (2.0)	1 (2.2)	39 (11.1)
Toxic plants	-	1 (1.1)	3 (4.5)	4 (8.5)	1 (2.9)	5 (9.8)	-	14 (4.0)
Cosmetic products	2 (12.5)	3 (3.3)	-	1 (2.1)	-	-	1 (2.2)	7 (2.0)
Other	1 (6.3)	4 (4.4)	1 (1.5)	-	-	-	4 (8.7)	10 (2.8)
Not informed	2 (12.5)	10 (10.9)	7 (10.4)	2 (4.3)	4 (11.8)	15 (29.4)	10 (21.7)	50 (14.2)
Total	16 (100)	92 (100)	67 (100)	47 (100)	34 (100)	51 (100)	46 (100)	353 (100)

Concerning the classification of medicines, the most frequent agents were anxiolytics (mainly benzodiazepines), followed by analgesics (8.5%) - among which paracetamol stands out (5.1%) - and antiepileptic drugs. The category “other” grouped the following medicines: antiasthmatics, hypoglycemic agents, contraceptives, antiemetics, antispastic drugs, antiparasitic agents, urinary tract antiseptics, antiulcer agents, bronchodilators, cardiotonic agents, nasal decongestants, inorganic mercury, ointments, erectile dysfunction drugs, hormonal supplements, finasteride, and vaccines.

The category of chemicals/cleaning products showed a greater percentage of accidental poisoning by sodium hypochlorite (chlorine and bleach) and sodium hydroxide (caustic soda). The hydrocarbon group included turpentine and kerosene, which have this component in their compositions.

Table 3 shows the distribution of the main products involved in accidental poisoning, according to the victims’ age group. Medicines presented the highest incidence in all ages, except in children aged one year; among them, chemicals were more prevalent. In this age group, pesticides and cleaning products were also significant.

**Table 3:** Categories and classes of products that caused accidental exogenous poisoning in the population aged zero to 19 years, treated at the Hospital João XXIII in Belo Horizonte, Minas Gerais, Brazil, 2013 (n=353).

	n	% per category	% of the total
Medicines	128	100	36.3
Anxiolytics	32	24.7	9.1
Analgesics/anti-inflammatory drugs	18	14.0	5.1
Antiepileptic drugs	10	7.8	2.8
Antipsychotics	8	6.2	2.3
Antimicrobial agents	8	6.2	2.3
Antidepressants	7	5.5	2.0
Antihistamines	5	3.9	1.4
Antihypertensive drugs	5	3.9	1.4
Vitamin supplement	5	3.9	1.4
Other	31	24	8.8
Chemicals and cleaning products	105	100	29.7
Sodium hydroxide	21	20.2	5.9
Sodium hypochlorite	21	20.2	5.9
Multiple chemicals	16	15.4	4.5
Hydrocarbon	7	6.6	2.0
Carbon monoxide	7	6.6	2.0
Ammonium hydroxide	6	5.8	1.7
Hydrochloric acid	4	3.9	1.1
Formaldehyde	4	3.9	1.1
Caustic	4	3.9	1.1
Other	15	14.3	4.0
Pesticides	39	100	11.0
Carbamate	9	23.1	2.5
Coumarin	8	20.5	2.3
Pyrethroid	8	20.5	2.3
Organophosphorus compound	4	10.3	1.1
Rodenticide	2	5.1	0.6
Tick spray	2	5.1	0.6
Other	6	15.4	1.7
Other categories	80	-	22.7

Forty-three patients (12%) needed to be hospitalized (length of stay higher than 24 hours). The length of stay ranged from 1 to 5 days, with an average of 1.6 and a median of 1.0 day.

The univariate analysis associated hospitalization with living in other cities, two or more products involved, and ingestion (Table 4). In the final multiple model, victims living in other cities (odds ratio - OR=5.20; 95% confidence interval - 95%CI 2.37-11.44) and those poisoned by two or more substances (OR=4.29; 95%CI 1.33-13.82) had a greater chance of hospitalization; it was not possible to include the variable route of exposure in the model, as all hospitalizations occurred in individuals exposed by ingestion.

**Table 4:** Univariate analysis of factors associated with hospitalization in cases of accidental poisoning treated at the Hospital João XXIII, Belo Horizonte, Minas Gerais, Brazil, 2013 (n=353).

Characteristic	Hospitalization n (%)		Crude OR (95%CI)	Adjusted OR (95%CI)	p-value
	No	Yes			
Gender					
Female	140 (45.2)	19 (44.2)	1.04 (0.54-1.97)		0.904
Male	170 (54.8)	24 (55.8)			
Age group					
3 years or younger	191 (61.6)	31 (72.1)	0.62 (0.30-1.25)		0.183
> 3 years	119 (38.4)	12 (27.9)			
Place of residence*					
Belo Horizonte	224 (81.2)	22 (55.0)	3.52 (1.76-7.04)	5.20 (2.37-11.44)	< 0.001
Other	52 (18.8)	18 (45.0)			
Local of exposure					
Home	279 (93.6)	38 (97.4)	0.38 (0.05-2.96)		0.343
Other	19 (6.4)	1 (2.6)			
Number of substances					
1	254 (93.7)	29 (80.6)	3.60 (1.38-9.42)	4.29 (1.33-13.82)	0.006
2 or more	17 (6.3)	7 (19.4)			
Substance type					
Medicine	109 (40.7)	20 (57.1)	1.94 (0.95-3.96)		0.064
Other	159 (59.3)	15 (42.9)			
Route of exposure					
Other	38 (13.1)	0 (0.0)		-	0.016
Ingestion	253 (86.9)	39 (100.0)			

*City of residence; OR: odds ratio; 95%CI: 95% confidence interval.

Out of the victims treated for accidental exogenous poisoning, only one died - a five-year-old who ingested propranolol, an antihypertensive drug. Three (0.9%) patients left the hospital facilities against medical advice without completing the treatment, and a single victim was transferred to another hospital.

## DISCUSSION

The results of this study show a predominance of treatments for accidental poisoning in male children, aged 0-4 years, particularly those aged 1-2 years. This finding corroborates the results from several studies conducted in different countries and scenarios.^8^ ^,^ ^9^ ^,^ ^10^ ^,^ ^11^ ^,^ ^12^ ^,^ ^13^ ^,^ ^14^

Soori^7^ And Manouchehrifar et al.^13^ found a higher incidence of cases among boys and in the age group 0-4 years, in studies carried out in Iran. A study conducted in India between 2004 and 2006 revealed that the mean age with the highest prevalence of accidental exogenous poisoning was 2-3 years.^14^ In Brazil, a study conducted at the Hospital Universitário de Maringá, Paraná,^10^ showed a higher number of poisonings among male children (52.2%) and in the age group 0-4 years (81.0%). Also in Brazil, in Cuiabá, a study indicated that most victims were male (60%) and aged 0-4 years (71%).^12^ These data point to the need for strict surveillance and protective measures targeted at younger children, as they are at higher risk of accidents, given their tendency to explore the environment where they live - an integral part of their cognitive and motor development. In the first years of life, children explore objects orally, facilitating the ingestion of toxic products.^5^ ^,^ ^15^ The combination of the need for discovery, oral exploration, and insufficient judgment of risks is characteristic of young children and can explain the predominance of accidental poisoning in this age group.

We underline the inversion of predominant gender with increasing age, that is, females were more prevalent in the age group over 12 years in this study. As intentional poisoning among adolescents is more frequent in females,^16^ these findings raise the suspicion that some cases have been wrongly classified as accidental in this age group. The fact that benzodiazepines were disproportionately involved in this age group corroborates this suspicion (5% of victims aged up to 12 years and 13% of those over this age).

Most (90%) accidents happened at home, corroborating findings from other national and international studies. Soori^7^ identified that 89% of poisonings in children occurred inside the house, especially in younger ones. In 75% of cases, toxic products were accessible to the victims. A study performed in Maringá^10^ also identified the victim’s home as the main place of poisoning (87% of cases), even when the child was under the protection and in the presence of an adult. The predominant triggering factors were easy access to medicines and exposure by ingestion.^10^ In the present study, information about the location within the residence where the accident happened was not available. However, in other reports,^5^ ^,^ ^17^ the living room, bedroom, and kitchen were the places with the most cases of poisoning in children.

In our study, the most common route of exposure was ingestion, which reached 82.7% of cases, a result largely corroborated by the literature.^10^ ^,^ ^14^ ^,^ ^18^ ^,^ ^19^ ^,^ ^20^

Our data showed that medicines were responsible for 36.5% of accidents, while other non-medicinal products (chemicals, pesticides, cleaning products, toxic plants, and cosmetics) accounted for 46.5% of cases. The predominance of medicines^7^ ^,^ ^10^ ^,^ ^12^ ^,^ ^13^ ^,^ ^19^ ^,^ ^20^ ^,^ ^21^ and cleaning products^12^ ^,^ ^18^ ^,^ ^22^ is a virtual constant in studies about poisoning in children.

In the present study, benzodiazepine anxiolytics represented the main group of medicines involved in poisonings. In a study by Anderson et al.,^23^ carried out in the United Kingdom, benzodiazepines represented 19% of poisoning cases, reinforcing that these substances often cause accidents. In an investigation by Azkunaga et al. (Spain),^20^ paracetamol held the first place (12.9%), followed by benzodiazepines (10.3%). Benzodiazepines are among the most used medicines in Brazil, considering all pharmaceutical classes,^24^ making them easily available in the domestic environment. Furthermore, we should consider the possibility that, in some cases, they were intentionally used, but this information was omitted in the report made at the time of treatment.

The category of substances involved and the child’s age showed some differences. In children <2 years old, the incidence of chemicals/cleaning products was higher, while poisoning by medicines was more prevalent in those older than two years. In the study by Brito and Martins,^12^ children under one year of age presented a greater number of cases of pesticide poisoning (66.6%); from one to four years, the main agents were cleaning products; and between five and nine years, pharmacological products (66.6%) had a higher incidence. Children’s easy access to different types of substances could be responsible for the different age patterns found.^5^ ^,^ ^21^ The chemical substances involved can also change according to availability. In rural areas, pesticides are usually involved.^18^ ^,^ ^25^ In places that require hydrocarbon fuels to produce energy and heat for homes, due to the relative scarcity of electric power supply, this group becomes more common.^22^ Thus, product availability at home and ease of access are clearly potential risk factors.

We highlight the incidence of poisoning caused by chemicals used in domestic activities (detergents, soap powder, bleach, drain cleaners); they can be found in most households in liquid form, are often inadequately stored, and their colorful aspect is attractive to children. In addition, these products are frequently sold in PET bottles without an identification label to inform their origin and provide instructions for safe use.^26^

Many accidents with caustic substances resulted from the domestic manipulation of these products. In some cases reported in this study, the caregiver declared having at home, at the time of the accident, a mixture made of different products, with caustic soda as the main ingredient, to prepare homemade soap. This practice can be dangerous, especially in households with young children and without the constant supervision of an adult.

Regarding pesticides, including carbamates, one of the main issues is their illegal trade for home use as a rodenticide. In Brazil, they are only legal for agricultural use.^27^

The effectiveness of adult supervision is hindered by how fast a child can get involved in an accident. Kouéta et al.^22^ and Tavares et al.^10^ did not identify a higher incidence of poisoning associated with a lack of supervision. In a control case study, Ramos et al.^5^ revealed that the risk fraction attributable to caregiver inattention was 13%, while for dangerous products stored at low heights, it represented 19%. These findings suggest that making the house safe by appropriately storing products out of the reach of children can be a more effective strategy.

In our study, only one victim died, resulting in a case fatality rate of 0.2%, which can be considered very low. In Colombia (2009), a study identified 187 deaths by poisoning, representing a mortality rate of 0.6 cases per 100 thousand inhabitants and a case fatality rate of 9.5 per 100 thousand poisoned patients.^28^ In a study by Kouéta et al.,^22^ 3% of cases progressed to death, of which 75% corresponded to poisoning of children aged one to four years. Death by medicine poisoning is more prevalent in children under four years of age and is usually accidental.^29^ The low case fatality rate observed in the present study could be associated with the fact that the treatment was carried out in a very well equipped reference unit, with professional teams that have extensive experience in the care of victims of poisoning. Moreover, most accidents involved only one substance, mainly medicines and cleaning products of low risk. As the interval between accident and treatment was not systematically recorded, we could not assess its impact on case fatality rate and length of stay.

Data from the present study show that 15% of the accidental poisoning in children and adolescents treated at the emergency care resulted in hospitalization; the length of stay of the vast majority was up to two days. In a study by Brito and Martins,^12^ 24.4% of the victims were hospitalized.

The World Health Organization and the United Nations Children’s Fund recommend substituting potentially toxic domestic products by similar ones with a more benign profile, as well as providing immediate care and specialized information as strategies to prevent child poisoning and reduce associated lesions.^1^

One of the limitations of our study is the use of secondary data, based on patient treatment forms, preventing us from controlling the quality of the information registered in the medical records. The study was based on hospital treatments, restricting the extrapolation of our findings to the community. On the other hand, we emphasize that we conducted the study in a referral hospital in accidents and violence, which favors the representativeness of the findings in clinical environments, that is, it allows the comparison with other similar services.

In conclusion, accidental exogenous poisoning was higher in children aged one to two years, males, and those who live in Belo Horizonte. The main causative agents of exogenous poisoning were medicines, followed by cleaning products and chemicals. Living outside Belo Horizonte and ingesting multiple substances were risk factors for hospitalization among victims who sought emergency care for poisoning. The pattern of products and substances involved, the age profile, and the predominance of poisoning by ingestion in the domestic environment indicate that opportunities for preventive actions might have been missed
